# Supplementary material for: Quantifying functional group compositions of household fuel-burning emissions
Source: Atmos Meas Tech. Author manuscript; Available in PMC 2025 Apr 22. (PMC11151727; doi:10.5194/amt-17-2401-2024)
Supplement: Supplement1 [file NIHMS1990583-supplement-Supplement1.pdf]

Supplement of Atmos. Meas. Tech., 17, 2401–2413, 2024
https://doi.org/10.5194/amt-17-2401-2024-supplement
© Author(s) 2024. CC BY 4.0 License.

Atmospheric Measurement Techniques
Open Access

Supplement of

Quantifying functional group compositions of household fuel-burning emissions

Emily Y. Li et al.

Correspondence to: Emily Y. Li (li.emily@epa.gov) and Satoshi Takahama (satoshi.takahama@epfl.ch)

The copyright of individual parts of the supplement might differ from the article licence.

Supplemental Information

Contents

**S1**	**WBT protocol**	**S2**
**S2**	**List of PAHs analyzed by GC-MS**	**S3**
**S3**	**Baseline correction**	**S4**
**S4**	**EC information in the FTIR spectra**	**S5**
**S5**	**Determination of influential functional groups for prediction**	**S6**
**S6**	**Influential group frequencies for OC**	**S7**
**S7**	**OC, EC, and $PM_{2.5}$ emissions supplement**	**S8**
**S8**	**Fuels Analysis, Original Fuel Compositions**	**S11**
**S9**	**Mid-infrared spectra of $PM_{2.5}$ separated by stove type**	**S12**
**S10**	**FG emission factors and corresponding absorption bands**	**S12**
**S11**	**Using out-of-plane vibrations for quantifying aromatic CH**	**S13**
**S12**	**Comparison of OC estimates**	**S15**

S1 WBT protocol

The WBT protocol used in this study is illustrated as a schematic in Fig. S1.

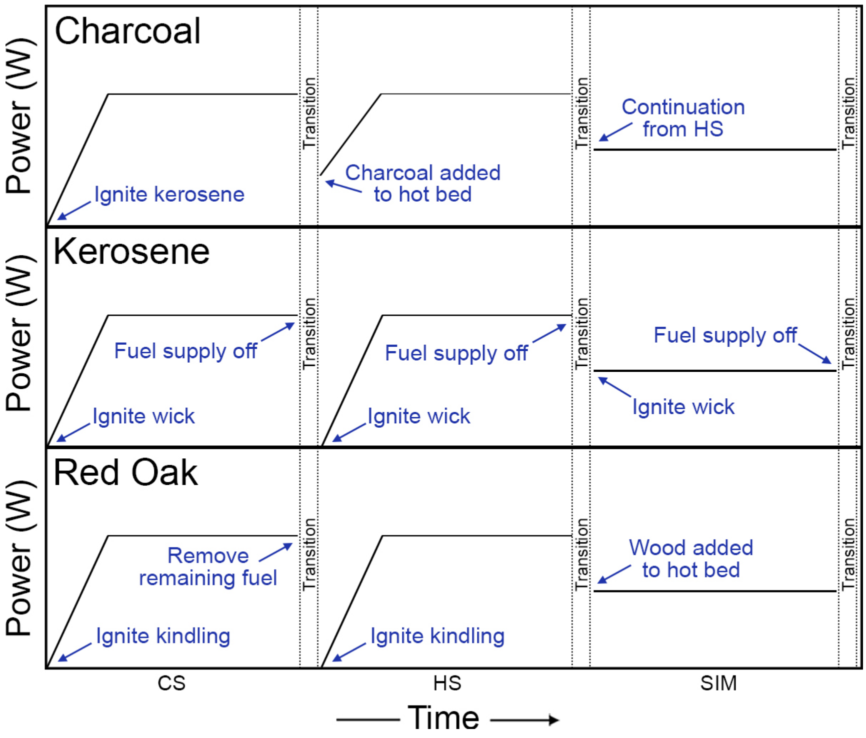

Figure S1: Schematic of WBT protocol for each fuel.

S2 List of PAHs analyzed by GC-MS

Table S1: List of PAHs with molecular formula and C/H molar ratios.

Compound	Molecular Formula	C/H molar ratio
Naphthalene	$C_{10}H_8$	1.25
1-Methylnaphthalene	$C_{11}H_{10}$	1.1
2-Methylnaphthalene	$C_{11}H_{10}$	1.1
2,6-Dimethylnaphthalene	$C_{12}H_{12}$	1
Acenaphthylene	$C_{12}H_8$	1.5
Acenaphthene	$C_{12}H_{10}$	1.2
Dibenzofuran	$C_{12}H_8O$	1.5
Fluorene	$C_{13}H_{10}$	1.3
Methylfluorene	$C_{14}H_{12}$	1.17
Phenanthrene	$C_{14}H_{10}$	1.4
Anthracene	$C_{14}H_{10}$	1.4
9-Methylanthracene	$C_{15}H_{12}$	1.25
Fluoranthene	$C_{16}H_{10}$	1.6
Pyrene	$C_{16}H_{10}$	1.6
Benzo(ghi)fluoranthene	$C_{18}H_{10}$	1.8
Cyclopenta(cd)pyrene	$C_{18}H_{10}$	1.8
Benz(a)anthracene	$C_{18}H_{12}$	1.5
Chrysene	$C_{18}H_{12}$	1.5
1-Methylchrysene	$C_{19}H_{14}$	1.36
Benzo(b)fluoranthene	$C_{20}H_{12}$	1.67
Benzo(k)fluoranthene	$C_{23}H_{16}$	1.44
Benzo(e)pyrene	$C_{20}H_{12}$	1.67
Benzo(a)pyrene	$C_{20}H_{12}$	1.67
Perylene	$C_{20}H_{12}$	1.67
Indeno[1,2,3-cd]pyrene	$C_{22}H_{12}$	1.83
Dibenzo[a,h]anthracene	$C_{22}H_{14}$	1.57
Benzo[ghi]perylene	$C_{22}H_{12}$	1.83
Coronene	$C_{24}H_{12}$	2

S3 Baseline correction

The procedure for baseline correction is introduced in Sec. 2.3.1. Figure S2 illustrates the raw spectrum, fitted smoothing spline, and residual spectrum for a single cookstove filter sample. The residual contains absorption peaks from desired analytes but also from the PTFE substrate (large peak at 1300–1000 cm^{-1}). A smoothing spline was similarly fitted to a raw blank filter spectrum, which was selected based on similarity of its PTFE peak intensity at 1150 cm^{-1} to the sample spectrum. The blank residual spectrum was further scaled to more closely match the intensity of the 1150 cm^{-1} peak of the sample residual spectrum. Thereafter, the scaled blank residual spectrum was subtracted from the sample spectrum (Figure S3).

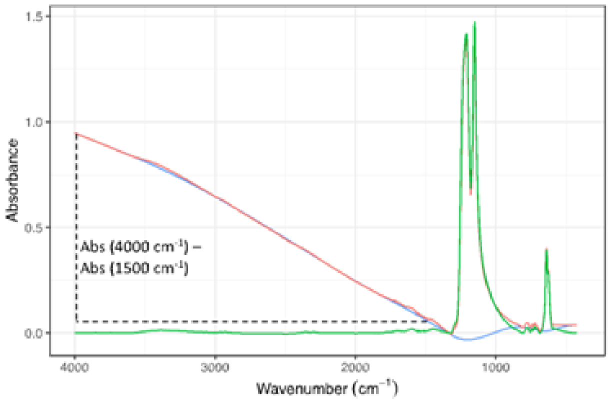

Figure S2: Raw spectrum (red), fitted smoothing splines baseline (blue), and baseline corrected spectrum (green) of a cookstove filter sample overlayed. The difference in absorbance of the baseline (blue) spectra (Fig. 1) between wavenumber 4000 cm^{-1} and 1500 cm^{-1} is related to the EC content in the sample (Section S4)

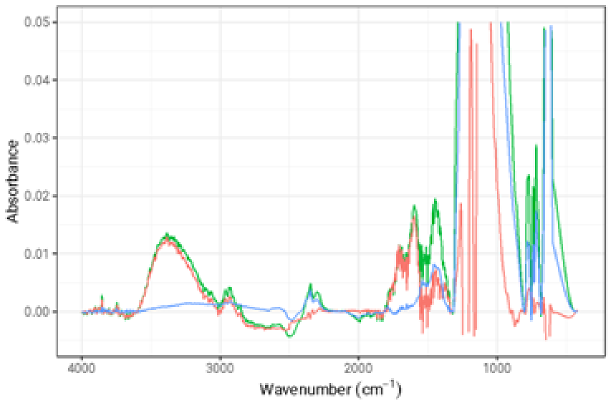

Figure S3: Baseline corrected sample spectrum (green), baseline corrected blank spectrum (blue), and final spectrum (red) for a cookstove filter sample.

S4 EC information in the FTIR spectra

The absorbance at each wavenumber is the sum of FG absorbances at that wavenumber, PTFE filter membrane light scattering, organic material light scattering, inorganic material light scattering and absorption of EC due to electronic transitions. In the current data set, inorganic salts are not present. Thus, outside of light scattering, only EC and OC change the absorbance. There are some variations in filter light scattering from sample to sample due to factors such as thickness variation, fiber orientation and filter porosity. As the filter scattering is believed to be the major absorbing component due to its substantial mass and thickness compared to particulate matter, initially, its variation should be eliminated. For this purpose, the highest C-F absorption peak was chosen. Each spectrum was baseline-corrected, which separated the spectrum in two parts. The first part, called the baseline-corrected spectrum, contains variations due to FG absorptions, which appear usually as sharp to semi-broad peaks. The second part contains low level gradual variations, which usually arise from light scattering of filter and particulate matter, and EC absorption. In order to eliminate filter contribution to background, a scaled version of an empty filter background spectrum was subtracted from the background part of each spectrum. During the scaling process, the absorbance at each wavenumber for empty spectrum was multiplied by a factor which made the highest peak equal to that of the desired spectrum. By regressing the slope of corrected background absorption to EC and OC we get a relatively high correlation coefficient $R^2 = 0.85$ (Fig. S4). The EC regression coefficient from the ordinary least squares solution is approximately 2.2 times that of OC or approximately four times that of OM.

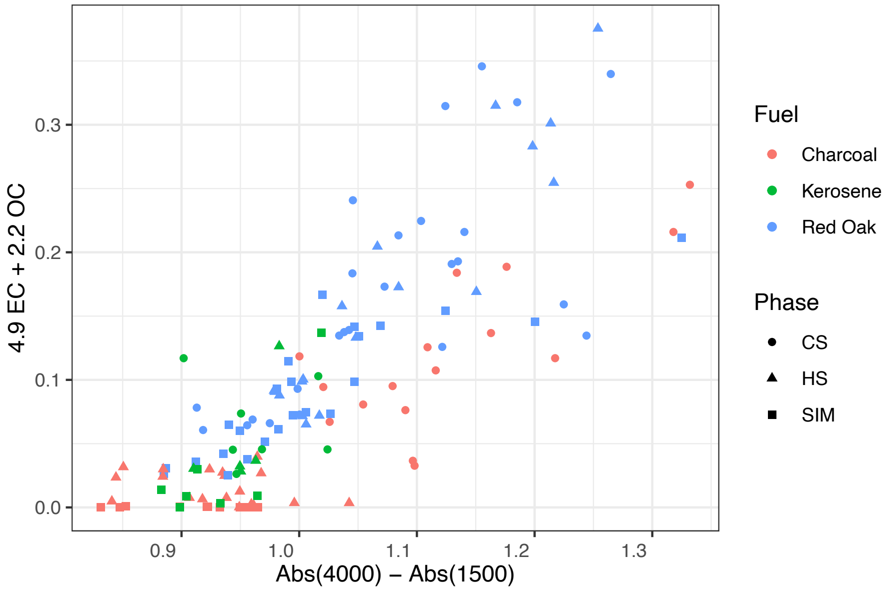

Figure S4: Baseline at 4000 cm^{-1} in the FTIR spectra regressed against EC and OC measured by TOT. The coefficients of the regression are shown on the vertical axis.

S5 Determination of influential functional groups for prediction

In order to identify the FGs that contribute the most to TOT OC, we statistically combined collocated TOT OC (on Qf filters) with FTIR (on PTFE filter) measurements. For this purpose, artifact-corrected TOT OC concentrations regressed against the baseline-corrected, blank-subtracted FTIR spectra using partial least squares regression (PLSR). Thereafter, influential FGs for OC were determined based on their VIP scores. By regressing TOT OC concentrations against FTIR spectra, we seek the solution of the following equation:

$$\mathbf{y} = \mathbf{X}\mathbf{b} + \mathbf{e}, \tag{S1}$$

where $\mathbf{X}$ (n×p) is the FTIR spectra matrix (n samples and p wavenumbers), $\mathbf{y}$ (n×1) is the vector TOT OC concentration, and $\mathbf{e}$ is the vector of residuals. We chose the univariate PLSR to solve the equation above (Wold et al., 1983). Univariate PLSR projects $\mathbf{X}$ onto basis $\mathbf{P}$ with orthogonal scores $\mathbf{T}$ and residual matrix $\mathbf{E}$ (Eq. S2) such that the covariance between each score column and $\mathbf{y}$ is maximized. In Eq. (S3), $\mathbf{b}$ is the regression coefficient of $\mathbf{y}$ as a function of scores ($\mathbf{T}$) and $\mathbf{f}$ is the vector of residuals.

$$\mathbf{X} = \mathbf{T}\mathbf{P}^T + \mathbf{E}, \tag{S2}$$

$$\mathbf{y} = \mathbf{T}\mathbf{b} + \mathbf{f}. \tag{S3}$$

After solving the regression equation using PLSR, a repeated 10-fold cross validation was applied to indicate the optimal number of latent variables. We used variable importance in projection (VIP) (Wold et al., 1993; Chong and Jun, 2005; Takahama et al., 2016), to identify the important FGs. The VIP score of the jth wavenumber is calculated by considering all h latent variables in the model as shown in Eq. (S4):

$$VIP_j = \sqrt{p\frac{\sum_{k=1}^{h} SS(b_k\mathbf{t}_k)(w_{jk}/\|\mathbf{w}_k\|)^2}{\sum_{k=1}^{h} SS(b_k\mathbf{t}_k)}}, \tag{S4}$$

where $SS(b_k\mathbf{t}_k) = b_k^2\mathbf{t}_k^t\mathbf{t}_k$. Since the average of squared VIP scores is equal to one, wavenumbers with VIP scores greater than one are considered to have higher-than-average contribution to the response variable (i.e., are influential).

We also followed a similar procedure to identify important bands correlated with the PAHs measured by GC-MS (Fig. 7).

S6 Influential group frequencies for OC

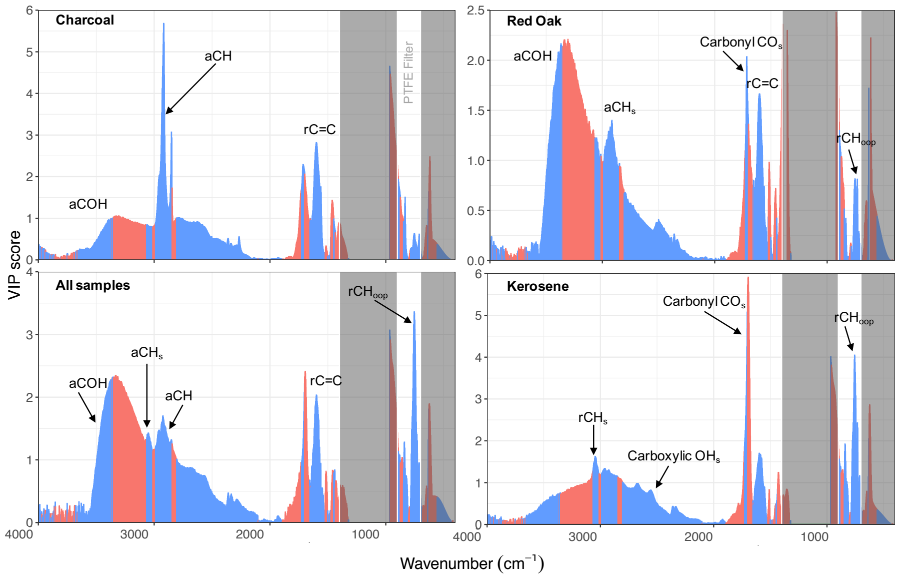

Figure S5: VIP scores of TOT OC regressed against baseline-corrected mid-infrared absorbances for each fuel type. Important group frequencies for each fuel are indicated. The regions with negative regression coefficients are shown in red, and regions with positive regression coefficients are shown in blue.

As seen in Fig. S5, the absorption region of the aCOH group in the 3500–3400 cm^{-1} range has the highest VIP scores for red oak combustion, suggesting this group is the the most influential FG for the OC emitted from red oak combustion. After aCOH, the carbonyl CO, aromatic ring C=C, and out-of-plane aromatic CH, and aCH regions have the highest VIP scores, respectively. As expected, although the inorganic nitrate peak is prominent in spectra of the oak burning aerosols at 1400 cm^{-1}, this group does not have either high VIP scores or positive regression coefficients of TOT OC. The greater-than-one VIP scores in the 3400–2400 cm^{-1} range are believed to indicate the alcohol group in sugar moieties, which are abundant in wood burning aerosols. The 3400–3100 cm^{-1} region, in which ammonium absorbs strongly, has high VIP scores with negative regression coefficients for all fuel. The PLSR models use the information from this region to correct the ammonium interference with organics.

For aerosols emitted from kerosene combustion, both the out-of-plane and the stretching bands of rCH have the highest VIP scores, indicating the importance of aromatic compounds for OC emitted from kerosene combustion. The carbonyl peak along with the region indicating the dimerized acid OH have also high VIP scores, suggesting that carboxylic acids are also important constituents of OC. Although aCH is the most important group in unburned kerosene, it does appear to be as important in OC emitted from kerosene combustion (i.e., no high VIP scores).

For aerosols emitted from charcoal combustion, the aCH region (3000–2800 cm^{-1}) has considerably higher VIP scores than other FGs, suggesting that the majority of OC is due to this functional group. Regions attributed to the aromatic ring C=C, carbonyl CO, and aCOH have, in order, the highest VIP scores after aCH.

When the PLSR model is developed using all samples instead of each fuel, the resulting VIP scores are similar to the average of previously calculated VIP scores for each fuel. However, the regression is pulled more toward those of red oak aerosols due to their higher OC emissions.

Based on the VIP scores, aromatic CH (rCH), alcohol COH (aCOH), aliphatic CH (aCH), aromatic C=C, non-acid carbonyl (naCO), and carboxylic acid COOH are the most important FGs in the OC emitted from the combustion of red oak, kerosene, and charcoal. Since FGs such as amine, organonitrate, and carboxylate absorb in the same region as the aromatic C=C, the quantification of C=C group is uncertain. Moreover, the absorption coefficient of C=C band is variable and is enhanced by the irregularity of aromatic molecules caused by different ring substitutions (Russo et al., 2014), adding to this uncertainty. We used the rCH FG as an alternative means to quantify C and H atoms of aromatic rings.

S7 OC, EC, and $PM_{2.5}$ emissions supplement

In Figure S6, the Butterfly model 2412 pressure-style kerosene cookstove displays relatively high emissions across all phases in three experiments. Higher than optimal emissions in these specific experiments were caused by a partially clogged burner orifice. A tool with a small wire is provided with the stove for cleaning the orifice, and the tool was used each time the orifice clogged. If the mentioned outlier samples are omitted, the average EC/TC ratio for kerosene is 0.44 ± 0.31 and appears to be in general lower in the SIM phase (Fig. S6).

As can be seen from Fig. S7, for the majority of samples, the sum of EC and OM (OM/OC $\times$ OC, OM/OC is calculated from FTIR measurements) constitutes the majority of $PM_{2.5}$ mass. This suggests that $PM_{2.5}$ is mostly composed of EC and OM and the contribution of inorganics is negligible. Previous studies suggest that EC and OM (estimated as OM/OC$\times$OC, where OM/OC is estimated from FTIR measurements) constitute the majority of the gravimetrically-measured $PM_{2.5}$ mass , and that inorganic salts (e.g., ammonium sulfate and ammonium nitrate) are believed to contribute negligibly to the emissions for the majority of samples(Roden et al., 2006). However, some wood and charcoal combustion $PM_{2.5}$ are considerably higher than the sum of EC and OM. For example, both the EcoZoom and Jiko Koa stoves have higher $PM_{2.5}$ and low EC during the SIM phase (Fig. S6) accompanied by a relatively lower MCE. These are among samples with relatively high inorganic nitrate absorbances in their FTIR spectra (Fig. S8). For wood and charcoal combustion samples in the $0.4 < (OM+EC)/PM_{2.5} < 0.7$ range, a clear inorganic nitrate peak is observed (Fig. S8).

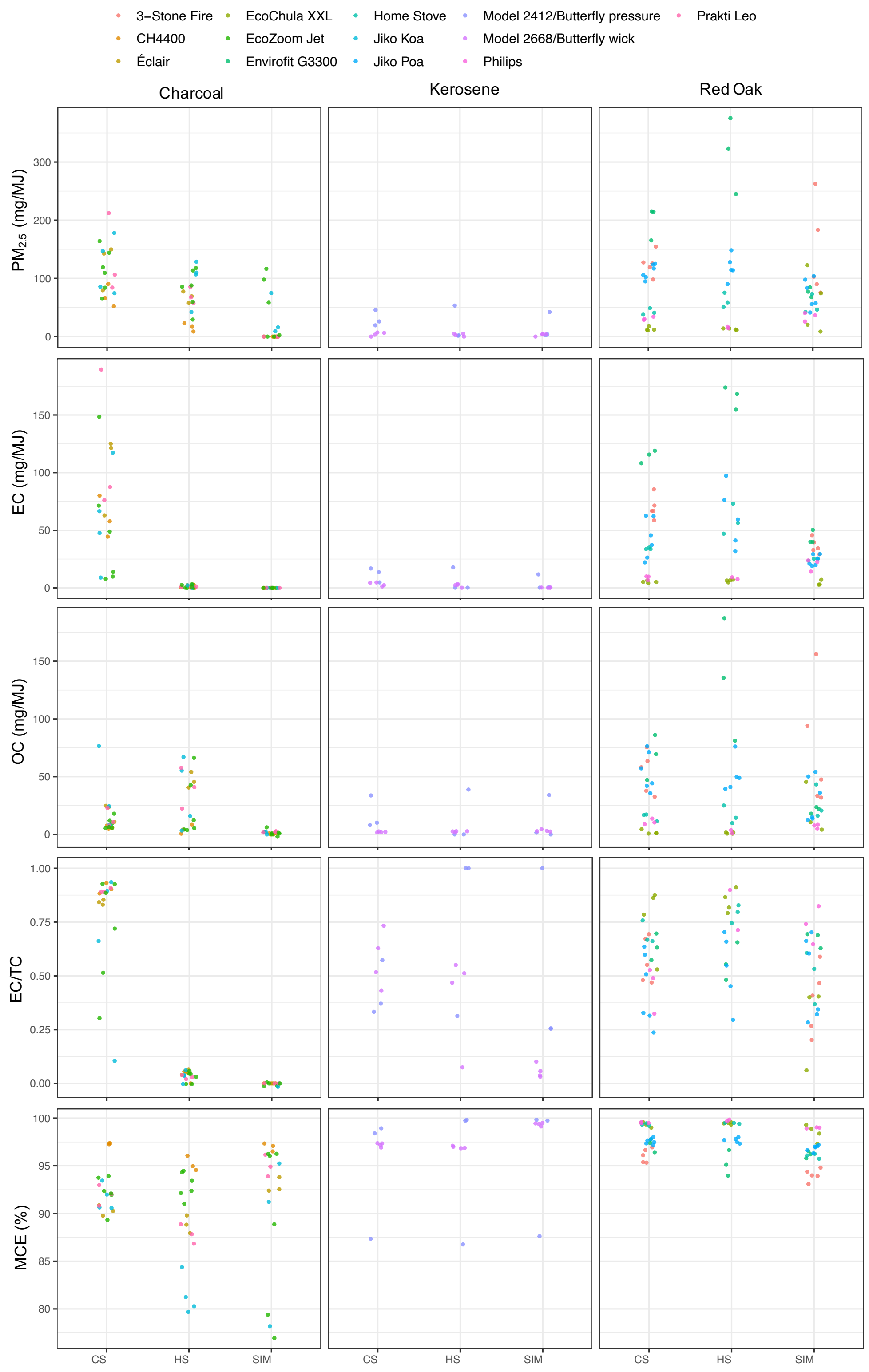

Figure S6: The emissions factors, EC/TC, and modified combustion efficiency (MCE; $(\Delta CO_2+\Delta CO)/\Delta CO_2$) separated by fuel type, test phase, and cookstove.

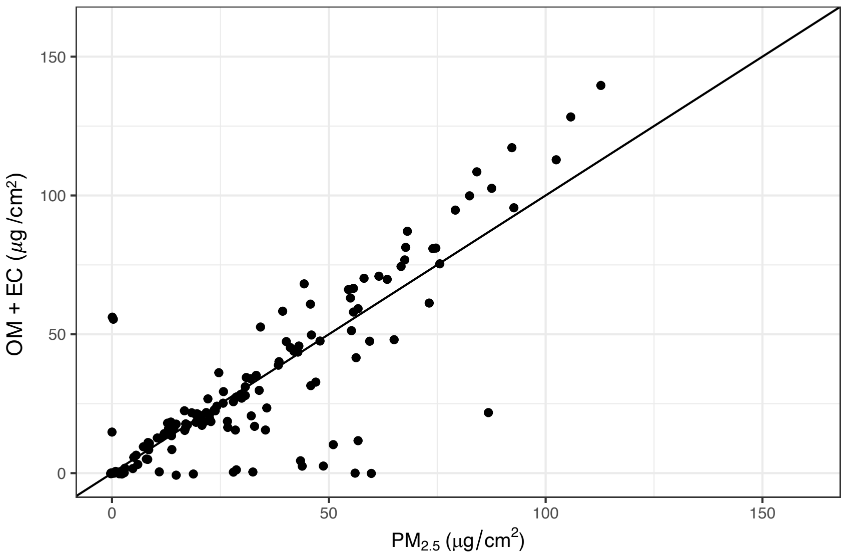

Figure S7: Scatter plot comparing $PM_{2.5}$ (gravimetric) measurements with the sum of artifact-corrected TOT OM (OM/OC × OC, where OM/OC is calculated from FTIR) and EC on quartz fiber filters.

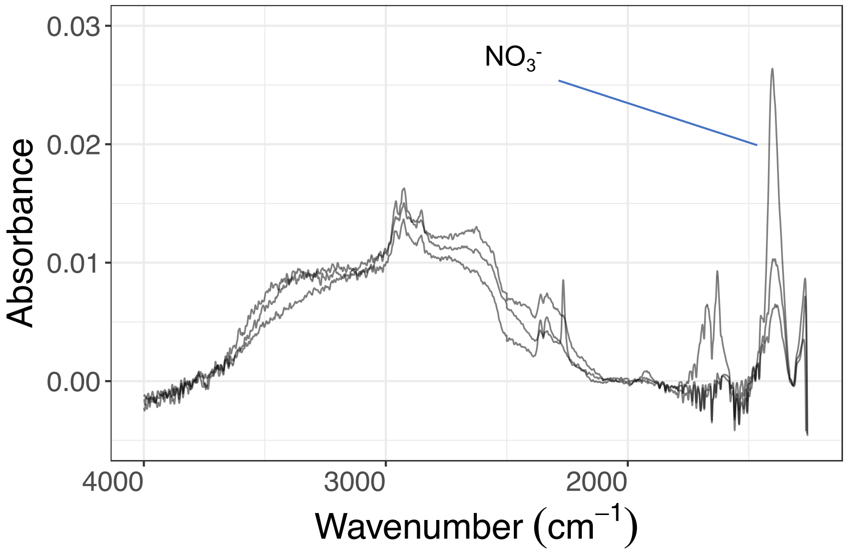

Figure S8: Spectra of three samples from charcoal particulate emissions with significant contributions of inorganic nitrate.

S8 Fuels Analysis, Original Fuel Compositions

Table S2: Original (pre-burn) fuel analysis.

Fuel	moisture	volatile	fixed carbon	ash	Sulfur	Carbon	Hydrogen	Nitrogen	Oxygen
Charcoal (as received)	2.95%	17.97%	77.32%	1.76%	0.02%	81.72%	3.04%	0.81%	9.70%
Charcoal (dry basis)		18.51%	79.67%	1.82%	0.02%	84.20%	3.13%	0.83%	10.00%
Kerosene					0.03%	85.93%	13.82%	0.01%	
Red Oak (as received)	5.28%	82.58%	11.78%	0.36%	0.08%	46.92%	5.74%	0.30%	41.33%
Red Oak (dry basis)		87.19%	12.44%	0.38%	0.08%	49.54%	6.05%	0.32%	43.64%
Alcohol					0.03%	36.34%	11.68%	0.03%	
LPG						82.09%	17.91%		

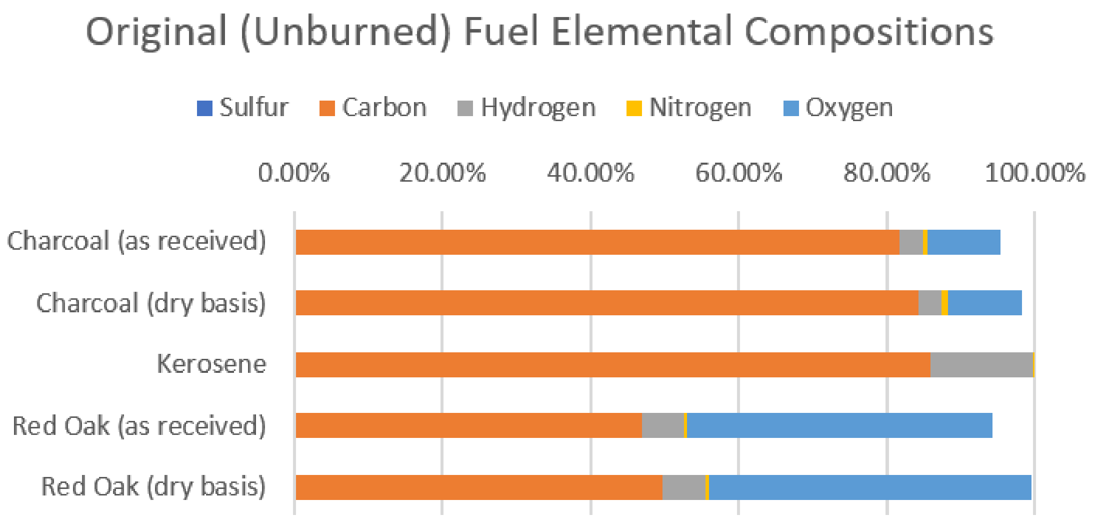

Figure S9: Elemental composition of unburned fuels. Numerical data shown in Table S2.

S9 Mid-infrared spectra of $PM_{2.5}$ separated by stove type

There are no systematic differences in spectra across stove types for red oak, but variations in overall emission factors are observed by stove type/experiment. Stove type matters for kerosene as well as charcoal. While spectroscopy features are similar, the magnitude of emissions can vary quite a bit. The burner type used (wick or orifice) appears to have a dominant effect on the composition of the emitted OM (Fig. S10).

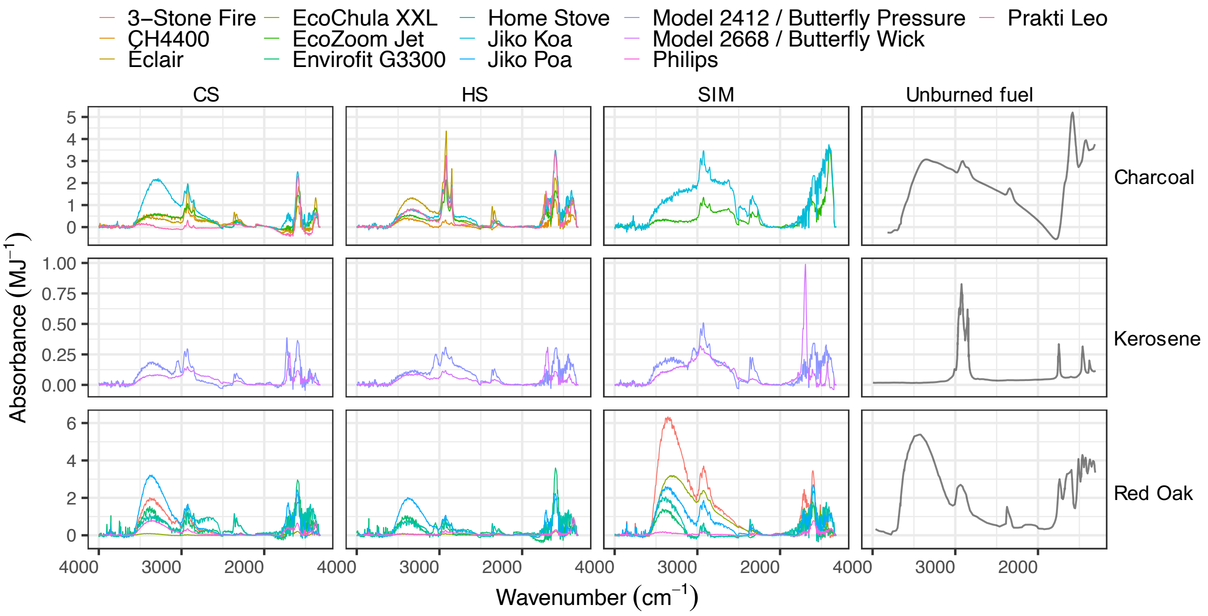

Figure S10: Average mid-infrared spectra of particulate emissions separated by source and phase and stove (normalized by energy delivered to the pot). CS = cold start, HS = hot start, SIM = simmering. Black spectra are for unburned fuels (wood, kerosene, and charcoal).

S10 FG emission factors and corresponding absorption bands

Table S3: Mean abundances in mg/MJ and spectral regions used for quantification. Regions are taken from Russell et al. (2009); Takahama et al. (2013); Ruggeri and Takahama (2016).

Group	Charcoal			Kerosene			Red Oak			Region (cm^{-1})
	CS	HS	SIM	CS	HS	SIM	CS	HS	SIM	
alcohol COH	6.52	10.27	16.37	1.94	2.3	2.79	13.65	9.02	18.39	3150–3670
alkane CH	8.73	16.94	16.94	1.91	1.77	2.43	10.67	7.97	15.2	2790–2930
na CO	0	0.09	0	0.04	0.09	0.23	0.15	0.11	0.08	1690–1750
carboxylic COOH	0.07	1.83	0.6	0.7	0.36	1.09	1.98	0.95	2.94	1690–1750, 2635–3200
aromatic CH	6.65	4.74	0	6.74	8.83	5.39	16.6	26.36	2.73	730–780

S11 Using out-of-plane vibrations for quantifying aromatic CH

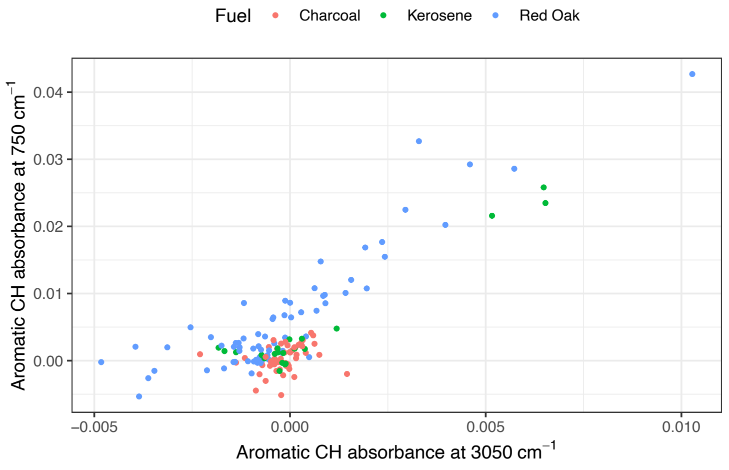

Figure S11: Scatter plot of aromatic CH stretching at 3050 cm^{-1} and out-of plane absorbances at 750 cm^{-1}.

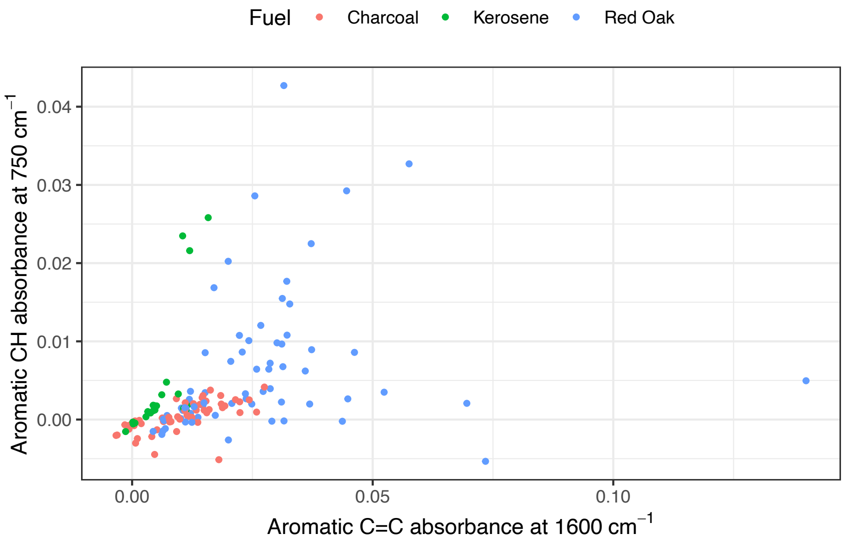

Figure S12: Scatter plot of aromatic C=C stretching at 1600 cm^{-1} and out-of plane absorbances at 750 cm^{-1}.

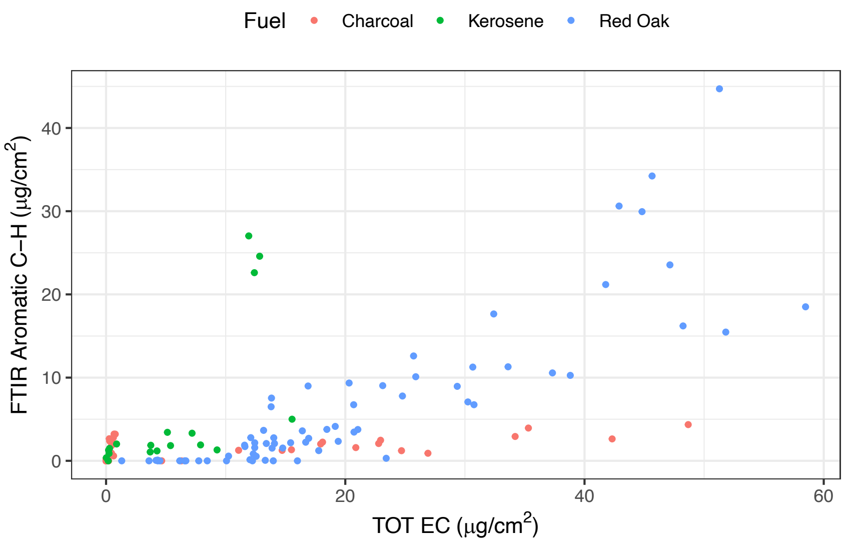

Figure S13: Scatter plot of aromatic CH concentration estimated using the peak at 750 cm^{-1} and TOT EC concentration.

S12 Comparison of OC estimates

FG OC estimated from sum of FGs is correlated with TOT OC measurements (R2 = 0.83), but predicts concentrations approximately 40% lower.

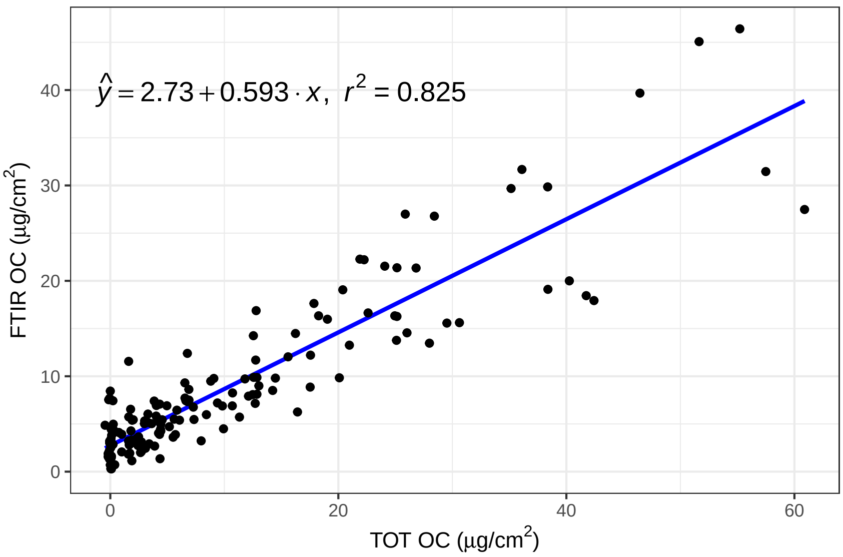

Figure S14: Scatter plot comparing OC (areal density on filters) estimated from TOT and FTIR.

References

Chong, I.-G. and Jun, C.-H.: Performance of Some Variable Selection Methods When Multicollinearity Is Present, Chemometrics and Intelligent Laboratory Systems, 78, 103–112, https://doi.org/10.1016/j.chemolab.2004.12.011, 2005.

Roden, C. A., Bond, T. C., Conway, S., and Pinel, A. B. O.: Emission Factors and Real-Time Optical Properties of Particles Emitted from Traditional Wood Burning Cookstoves, Environmental Science & Technology, 40, 6750–6757, https://doi.org/10.1021/es052080i, 2006.

Ruggeri, G. and Takahama, S.: Technical Note: Development of Chemoinformatic Tools to Enumerate Functional Groups in Molecules for Organic Aerosol Characterization, Atmospheric Chemistry and Physics, 16, 4401–4422, https://doi.org/10.5194/acp-16-4401-2016, 2016.

Russell, L. M., Takahama, S., Liu, S., Hawkins, L. N., Covert, D. S., Quinn, P. K., and Bates, T. S.: Oxygenated fraction and mass of organic aerosol from direct emission and atmospheric processing measured on the R/V Ronald Brown during TEXAQS/GoMACCS 2006, Journal of Geophysical Research-atmospheres, 114, D00F05, https://doi.org/10.1029/2008JD011275, 2009.

Russo, C., Stanzione, F., Tregrossi, A., and Ciajolo, A.: Infrared Spectroscopy of Some Carbon-Based Materials Relevant in Combustion: Qualitative and Quantitative Analysis of Hydrogen, Carbon, 74, 127–138, https://doi.org/10.1016/j.carbon.2014.03.014, 2014.

Takahama, S., Johnson, A., and Russell, L. M.: Quantification of Carboxylic and Carbonyl Functional Groups in Organic Aerosol Infrared Absorbance Spectra, Aerosol Sci. Tech., 47, 310–325, https://doi.org/10.1080/02786826.2012.752065, 2013.

Takahama, S., Ruggeri, G., and Dillner, A. M.: Analysis of Functional Groups in Atmospheric Aerosols by Infrared Spectroscopy: Sparse Methods for Statistical Selection of Relevant Absorption Bands, Atmospheric Measurement Techniques, 9, 3429–3454, https://doi.org/10.5194/amt-9-3429-2016, 2016.

Wold, S., Martens, H., and Wold, H.: The Multivariate Calibration Problem in Chemistry Solved by the PLS Method, in: Matrix Pencils, edited by Kågström, B. and Ruhe, A., Lect. Notes Math., pp. 286–293, Springer Berlin Heidelberg, 1983.

Wold, S., Johansson, E., and Cocchi, M.: 3D QSAR in Drug Design: Theory, Methods and Applications, ESCOM, Leiden, Holland, pp. 523–550, 1993.
